# Ultrathin ALD Aluminum Oxide Thin Films Suppress the Thermal Shrinkage of Battery Separator Membranes

**DOI:** 10.1021/acsomega.2c06318

**Published:** 2022-11-30

**Authors:** Leonardo Pires da Veiga, Colin Jeanguenat, Fabiana Lisco, Heng-Yu Li, Sylvain Nicolay, Christophe Ballif, Andrea Ingenito, Juan Jose Diaz Leon

**Affiliations:** † Centre Suisse d’Electronique et de Microtechnique SA, Sustainable Energy Center, Neuchâtel2002, Switzerland; ‡ Ecole Polytechnique Fédérale de Lausanne, PV-Lab, NeuchâtelCH-2000, Switzerland

## Abstract

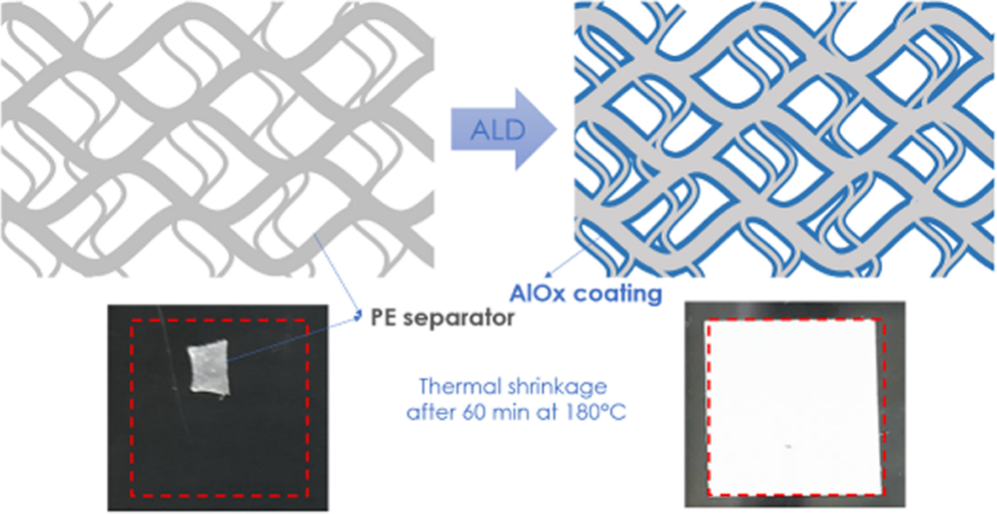

Thermal runaway is a major safety concern in the applications of Li-ion batteries, especially in the electric vehicle (EV) market. A key component to mitigate this risk is the separator membrane, a porous polymer film that prevents physical contact between the electrodes. Traditional polyolefin-based separators display significant thermal shrinkage (TS) above 100 °C, which increases the risk of battery failure; hence, suppressing the TS up to 180 °C is critical to enhancing the cell’s safety. In this article, we deposited thin-film coatings (less than 10 nm) of aluminum oxide by atomic layer deposition (ALD) on three different types of separator membranes. The deposition conditions and the plasma pretreatment were optimized to decrease the number of ALD cycles necessary to suppress TS without hindering the battery performance for all of the studied separators. A dependency on the separator composition and porosity was found. After 100 ALD cycles, the thermal shrinkage of a 15 μm thick polyethylene membrane with 50% porosity was measured to be below 1% at 180 °C, with ionic conductivity >1 mS/cm. Full battery cycling with NMC532 cathodes demonstrates no hindrance to the battery’s rate capability or the capacity retention rate compared to that of bare membranes during the first 100 cycles. These results display the potential of separators functionalized by ALD to enhance battery safety and improve battery performance without increasing the separator thickness and hence preserving excellent volumetric energy.

## Introduction

Electrochemical energy storage is a key technology for the global electrification of energy systems. Among different technologies, lithium-ion secondary battery has achieved widespread penetration in consumer electronics and, in the past decade, also in electrified transportation. Thanks to recent developments and the cost reduction driven by mass production,^1^ electric and hybrid mobility based on Li-ion batteries represent the most relevant effort to reduce carbon emissions in the private transport sector when using electricity with a low CO_2_ content.^2^

The Li-ion battery technology takes advantage of intercalation-based lithium-doped multimetal oxides, e.g., LiCoO_2_, LiMn_2_O_4_, and LiNiMnCoO_2_ as a positive electrode or cathode,^3^ a separator consisting of a porous membrane soaked in a Li-ion conducting liquid electrolyte to prevent electrical contact between the anode and cathode, and an intercalation host, like graphite or Li_2_TiO_3_ as a negative electrode or anode, respectively.^4^ Cathodes are chosen to have relatively high potential (3.7–4.3 V) vs Li/Li^+^, allowing for exceptionally high power and energy density compared to other electrochemical storage systems.^5^

Despite the technology being inherently safe with a failure rate estimated at around one for five million cells,^6^ thermal runaway and subsequent overheating are one of the major failure mechanisms, especially in high-power applications. In this context, the separator is a key component to improve lithium-ion batteries’ safety. The separator membrane prevents electrical contact between two electrodes (which would cause high-current and local overheating) while allowing for ionic conduction through it. Typically, polyolefin materials, such as porous polyethylene (PE) or polypropylene (PP) are used in liquid electrolyte Li-ion batteries because of their low cost and wide chemical stability. However, under mechanical, thermal, or operational abuse, PE separator membranes shrink as the cell’s temperature increases above 100 °C, thus approaching the melting temperature of PE. After shrinkage, the physical contact of positive and negative electrodes can no longer be prevented, causing an irreversible thermal runaway, swelling, and possible explosion.^7^ A typical solution to mitigate this issue consists in laminating a PP layer on both sides of the PE separator (PP/PE/PP) and making the battery nonoperational before the thermal shrinkage (TS) of the separator, hence preventing overheating.^8^ Despite this feature, it has been demonstrated that the shutdown function may not be enough to stop the thermal runaway, especially if the cell’s temperature quickly reaches the melting point of PP.^9^ To enhance the safety of the battery, a separator with no thermal shrinkage above 180 °C is desirable.^10^

Alternatively, ceramic- and polymer-based slurry coatings have been investigated to improve the thermomechanical properties and electrochemical performance of the polyolefin separators.^11^ Among these strategies, ceramic particles on polyolefin separators showed significant improvements in thermal stability and wettability while preserving flexibility and low manufacturing cost.^12^ However, a major drawback of this approach is the large increase in the separator thickness and the increase of inactive weight, leading to battery performance degradation, such as faster capacity fading and lower capacity retention at high charging rates (C-rates). The deposition uniformity and the agglomeration tendency of the ceramic particles blocking the pores are also often reported as downsides of this strategy. The above drawbacks are even more pronounced for double-side coated separators, which yield improved thermal stability by preventing bending at high temperatures (>120 °C).^13,14^

To overcome these limitations, coating of the separator by atomic layer deposition (ALD) has been used due to its self-limiting, conformal (even in the case of three-dimensional, 3D structures), and monolayer deposition properties.^13−16^ In 2012,^17^ Jung et al. showed how ALD of aluminum oxide (AlO*_x_*) on a commercial PP separator can allow for a drastic reduction of the TS and an increase in wettability without any noticeable thickness increase or flexibility loss. Despite these encouraging results, coatings on polyolefin separators using ALD are challenging due to the lack of intermolecular interactions between the membrane and the coating precursors, leading to a nonefficient subsurface nucleation mechanism. To mitigate the limitation of subsurface nucleation, Xu et al.^18^ demonstrated the potential of air plasma to activate the surface of the separator. The authors also observed an improvement in terms of conformality, which was used by Chen et al.^19^ to rationalize the higher thermal stability of plasma-activated ALD-coated membranes over the nonactivated ones.

To tackle the problem of pore narrowing also observed in the first ALD applications, Shen et al.^20^ took advantage of the large pore size of a nonwoven mat, which was combined with AlO*_x_* deposited by ALD. This approach demonstrated the expected improvements in terms of electrolyte uptake and ionic conductivity, leading to better rate capability and suppression of TS up to 270 °C. AlO*_x_* ALD on PE separators was also used by Moon et al.^21^ to enhance the uniformity of polydopamine coatings, which has been one of the bottlenecks of this chemical treatment since its origin.^22^ These examples show the ability of ALD coatings to reinforce the porous membrane without increasing its total thickness while avoiding pore clogging or the use of solvent slurries, in contrast with solution-based coatings.^23^ Despite its high potential, ALD is not widely used in the battery manufacturing chain due to high CapEx and OpEx and its low throughput, especially for films thicker than 10 nm. This can be mitigated using spatial ALD (compatible with fast roll-to-roll processing)^24,25^ and depositing ultrathin films (<10 nm). So far, there are no reports of effective thermal shrinkage suppression on standard PE separators using only ALD coatings at meaningful thicknesses of <10 nm.

Herein, we demonstrate a unique combination of an in situ plasma activation step and an optimized AlO*_x_* ALD process to suppress TS while maintaining or even improving the ionic conductivity of the separator. We show a process optimization leading to thin-film coatings (with less than 100 ALD cycles) that can be optimized for different porosities typical of PE separators and PP/PE/PP laminates, showcasing the versatility of this process. The correlation between the separator material, its pore size, and its porosity with the minimum film thickness needed to suppress TS is found and discussed. The results are presented starting from TS results, which reveal insights into the difference in coating growth depending on the membrane’s porous morphology. Water contact angle measurements are used to reinforce the conclusion proposed from the TS test. Gas permeability and ionic conductivity data show a relationship with water wettability quantified by water contact angle (WCA). Finally, coin cell cycling curves are presented to demonstrate how thin-film coatings on the separator can impact the battery cycling performance.

## Materials and Methods

### Separators

Different separators purchased from industrial suppliers were used in this work (see Table 1): a trilayer PP/PE/PP separator from Celgard (H2512), a PE48% porosity (PE48) separator from Liaoyuan Hongtu LIBS Technology Co., and a PE83% (PE83) from Lydall. The samples were cut into 10 × 10 cm^2^ squares before deposition and washed with 2-propanol.

**Table 1:** Structural Specifications of Polymer Separators before ALD Deposition

	material	thickness (μm)	porosity (%)	av. pore size (μm)	Gurley JIS (s)
Trilayer^27^	PP/PE/PP	25	39/44/39	0.028	320
PE48^28^	PE	16	48	<1	125
PE83^29^	PE	20	83	0.7	NA

### Materials

Graphite and NMC532 (MTI Corp, Richmond, CA) were used as negative and positive electrodes, respectively. A 1M LiPF6 mixture of EC/DMC/DEC 1:1:1 (Merck) was used as an electrolyte.

### Atomic Layer Deposition

Separator sheets were coated on both sides by thermal atomic layer deposition (ALD) of aluminum oxide using trimethylaluminum (TMA) and water as precursors with an Oxford FlexAL ALD system. The chamber temperature was 80 °C. The growth rate was measured to be 1.1 ± 0.1 Å/cycle on crystalline silicon witness samples. According to Wilson et al.,^26^ the thickness attained on polymers is similar to the one measured on Si. Since the exact thickness on top of membranes was not measured, cycles (and not thickness) were used throughout the text.

### Thermal Shrinkage Measurements

To evaluate the thermal stability of the composites, 2 × 2 cm^2^ squares were cut and sandwiched between two paper sheets. The TS measurement was taken in the machine direction starting from a temperature of 120 °C and then repeated 60 min after increasing the temperature sequentially by 20 °C. Equation 1 was used for the TS calculation
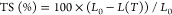
1where *L*_0_ is 2 cm and *L*(*T*) is the measured value along the machine direction at a given temperature.

### Contact Angle

Water contact angle (WCA) measurements were performed on DSA30 (Kruss) with a 2 μl drop.

### Microstructural Characterization

The morphology of the separators was observed with a JEOL-JSM-7500 TFE scanning electron microscope (SEM).

### Gas Permeability Measurements

The Gurley value of the bare and coated separators was measured using a Gas Permeameter GP-101A-G-T200 by Porous Materials Inc., situated at Ruschlikon’s IBM Research Institute Facility, belonging to ETH Department of Information Technology and Electrical Engineering.

### Battery Assembly

CR2032 coin cells were assembled in an argon-filled glovebox with oxygen and water level below 0.2 ppm. For ionic conductivity, the separators were sandwiched between two stainless steel spacers of 0.5 mm thickness and 15 mm diameter. The cell’s stack was sandwiched between two 0.5 mm spacers, held in place by a conical spring.

### Electrochemical Characterization

Coin cells were tested on a BCS815 cycler by Biologic. For EIS measurements, a 10 mV sinusoidal amplitude was applied from 10 kHz to 0.1 Hz. A Debye circuit was used to estimate the circuit’s resistance.

## Results and Discussion

**Figure 1:**
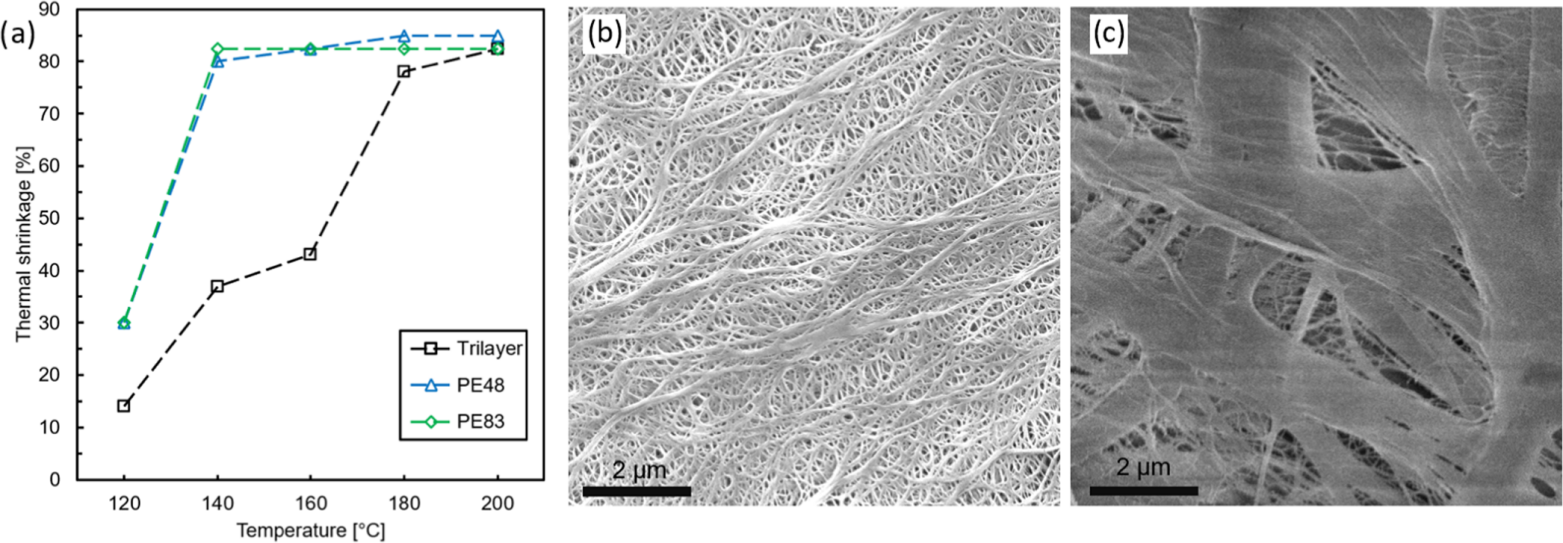
(a) Thermal shrinkage of bare polymer separators as a function of temperature after 30 min exposure. Top-view SEM showing the different microporous structures of (b) PE48 and (c) PE83.

To understand the dependence of the AlO*_x_* coating on the separator’s porosity, pore size, and polymer type, three different membranes were used: a trilayer separator composed of a PE core laminated with PP shells on both sides (Trilayer) and two pure PE membranes, with either 48 or 83% porosity (PE48 and PE83, respectively). Table 1 presents the thickness, porosity, average pore size, and Gurley value (gas permeability) of the three membranes from their respective specification sheets. These membranes were first characterized in terms of TS and microstructural properties. Figure 1a shows the TS of the different membranes described in Table 1 versus the annealing temperature. Figure 1b,c shows the SEM micrographs of the membranes visually highlighting the difference in structural morphology. PE48, displayed in Figure 1b, has a similar porosity to the PE core of Trilayer, just 4% higher, but it is 9 μm thinner; therefore, the Gurley value is lower. In other words, the increase in the porosity and the decrease in the thickness make PE48 more permeable to gas flow. An SEM image of PE83 is presented in Figure 1c. This membrane was chosen for its exceptionally high porosity and large average pore size; in fact, its permeability is so high that it cannot be evaluated by the Gurley JIS, thus representing the opposite extreme to Trilayer. The TS of porous polyolefins depends mostly on the melting point of the polymer matrix. For pure PE, the phase change occurs between 120 and 140 °C, which correlates with a steep increase in TS as observed in Figure 1a for PE48 and PE83. Given the seemingly complete overlap of the TS curves of these two materials, it can be concluded that the morphology of the porous structure does not significantly influence the thermal integrity of the PE monolayers. The specific design of the trilayer separators, with the two outer layers being made of PP, yields two steps in the TS curve, coinciding with the melting points of PE and PP, the latter being reported to be between 170 and 180 °C (Figure 1a, black). To evaluate the impact of the AlO*_x_* coating on the TS, we evaluated the latter at 180 °C, as this temperature is safely above both melting points and is the temperature at which runaway starts.

**Figure 2:**
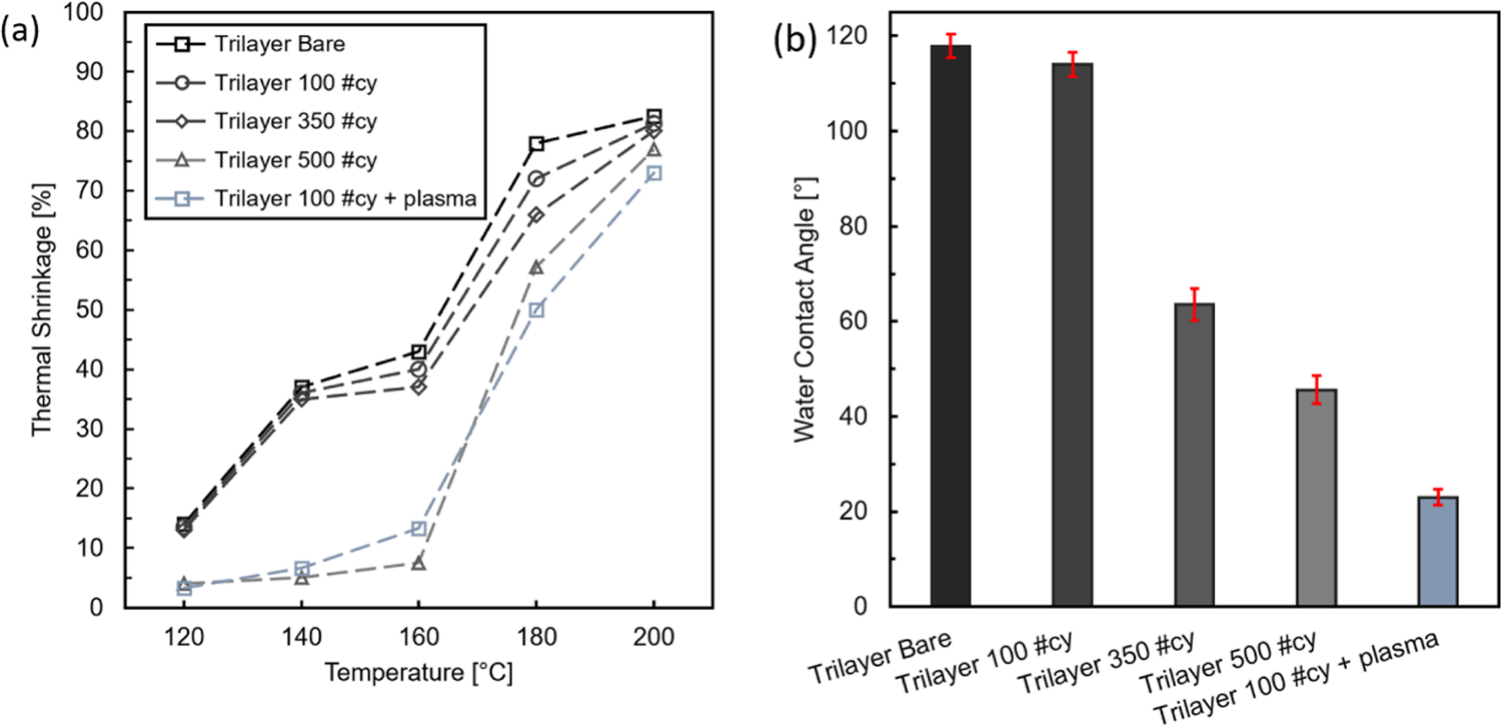
(a) Thermal shrinkage of trilayer separators as a function of the temperature of trilayer separator for different amounts of ALD cycles after 30 min exposure at a given temperature and (b) static water contact angle of the same samples measured 1 s after the deposition of a 2 μL drop.

First, ALD AlO*_x_* coatings were applied to the trilayer separator, since it is supposed to have better thermal integrity due to the PP layers. An N_2_/O_2_ plasma pretreatment was introduced to improve the conformality of the ALD coatings on the membranes. To evaluate the impact of the plasma treatment, trilayer separators were coated with AlO*_x_* ALD layers with or without the pretreatment. Figure 2a shows the TS versus temperature, while Figure 2b displays the WCA for trilayer separators coated with a different number of ALD cycles (#cy). Significant suppression of TS by deposition of the AlO*_x_* shell can be achieved for the trilayer separator without or without plasma pretreatment, but at a different number of #cy (Figure 2a). For the nontreated samples, the TS decreases by 5% after 350 #cy and is suppressed up to 160 °C only after 500 #cy. The WCA of the same samples (Figure 2b) reveals that 350 #cy are sufficient for halving the WCA from 120 to 60°, and the angle reaches 47° after 500 #cy. This shows that AlO*_x_* covers the surface of the PP layer, thus decreasing its interface energy with water, and that the coverage progresses with the increasing #cy.

Figure 2a shows how pretreating the sheets with an N_2_/O_2_ plasma significantly reduces the number of #cy required to suppress TS at 160 °C from 500 to 100 #cy. A lower WCA is also measured, indicating an improvement in surface coverage and/or conformality. This could be a consequence of the plasma improving the germination of AlO*_x_* islands in the first ALD cycles, increasing the germination density.^30,31^ Despite this improvement, it is not feasible to yield a total suppression of the TS after the melting point of PP (∼170–180 °C), suggesting that a structurally stable shell was not built around the PP matrix. Even though the WCA measurements show that the PP surface is covered by the AlO*_x_* coating, it was not possible to prevent the structure to fail once its melting point is reached. This could suggest either that the ALD coating is more effective on PE than PP with equivalent coating thickness, or that the growth of AlO*_x_* is hindered on PP. Both hypotheses are possible since the membranes are different in chemical composition, mechanical proprieties, and porous structure. These investigations allow nonetheless to indicate a positive impact of the AlO*_x_* coating on the TS properties of the trilayer membrane.

**Figure 3:**
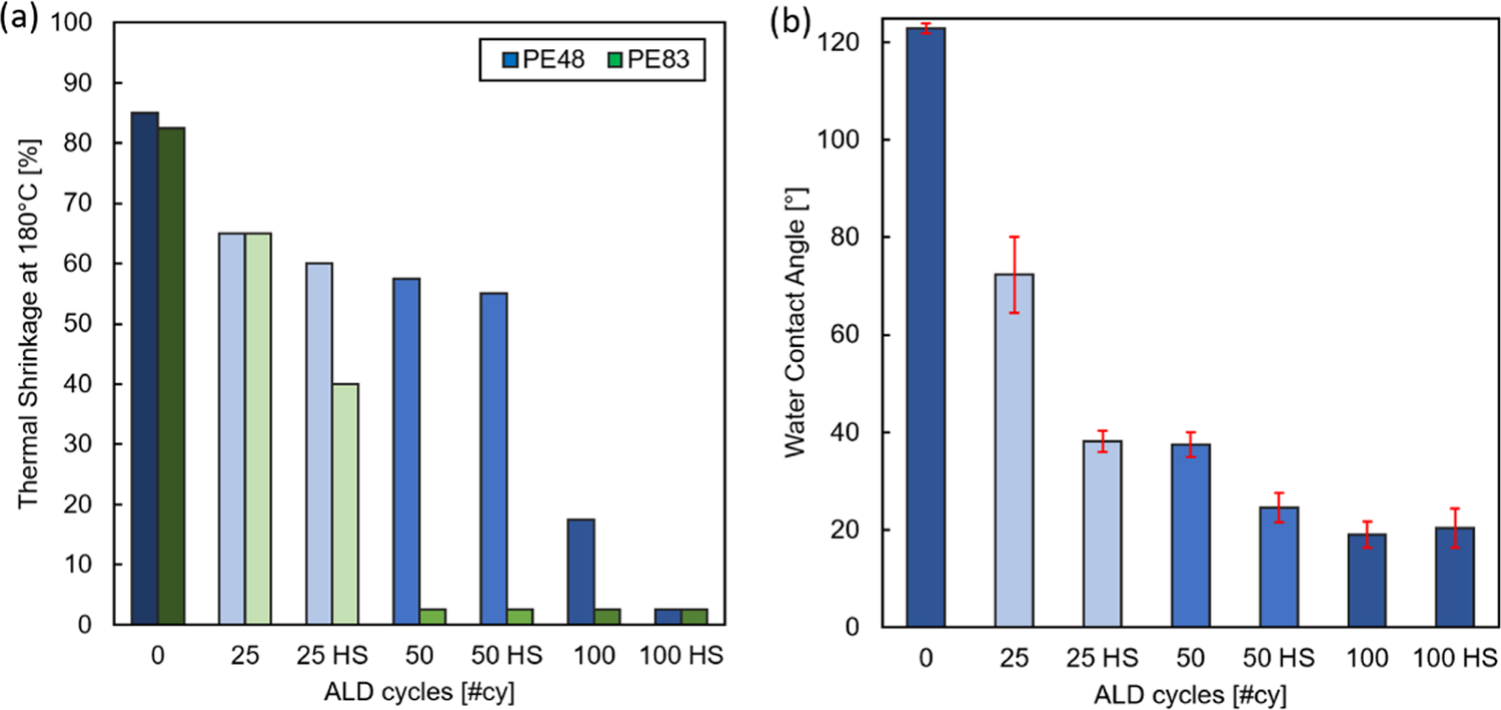
(a) Thermal shrinkage of PE separators coated by ALD after 30 min exposure at 180 °C and (b) static water contact angle of PE48 separator for the increasing number of ALD cycles with hold step and angle measured 1 s after deposition of a 2 μL drop. The data is an average of four measurements per side. HS indicates a hold step after dosing and before purging the ALD precursors.

To better understand the TS properties of AlO*_x_* ALD on PE, ALD deposition was performed on pure PE membranes with higher permeability (PE48 and PE83, see Table 1). In this case, a plasma pretreatment was used for all samples. Furthermore, a hold step (HS) was introduced in the recipe after each precursor step to explore whether the residence time of the precursors during ALD influenced the coating scaffolding properties. A comparison of the EDX spectra of bare PE48 and 50 #cy PE48 can be found in Figure S1 in the Supporting Information, where the appearance of the characteristic peaks of oxygen and aluminum is reported, confirming the presence of an AlO*_x_* layer after 50 #cy. Figure 3a shows the TS of PE48 and PE83 membranes after 25, 50, and 100 #cy with and without HS. For bare PE separators, there is little to no variation in TS from 140 to 200 °C, since PP is absent (as opposite for Trilayer in Figure 2), so the TS value was shown only at 180 °C for simplification. Figure 3b shows the WCA for PE48 membranes with different #cy (the WCA of PE83 is not displayed because the water droplets are absorbed by the membrane before a reproducible measurement can be performed). Pictures of the TS and the WCA tests can be found in Supporting Information Figures S2 and S3, respectively. As previously observed, both PE48 and PE83 bare membranes shrink fully at 180 °C and their WCA is close to 120 °C. The first 25 #cy reduce the WCA of PE48 to 72°; however, if the cycles are performed with HS, the WCA decrease is more marked, going below 40°. Already, the impact of membrane composition is noticeable: the Trilayer’s WCA only reaches 45° after 500 #cy, while PE48 with plasma pretreatment and HS requires only 25 #cy. The WCA for 50 #cy then reaches similar values to that for 25 #cy with HS, while that for 50 #cy with HS decreases further to an average of 25°. After that, no significant decrease in WCA is measured. This shows that both the plasma pretreatment and the HS contribute to improving the surface coverage of the PE pores. PE48 reaches 40° WCA after 50 #cy or 25 #cy with HS. It is hypothesized that after 25 #cy in the absence of the HS, the islands are not large enough to fully cover the PE surface; however, with HS, the island population is denser and thus covers a larger fraction of the PE surface. ALD is a technique that theoretically allows growing a monolayer per cycle; therefore, to accelerate surface coverage, one must increase the density of germination sites. Knowing that the germination of AlO*_x_* depends on the formation of subsurface islands,^32,33^ the plasma pretreatment could improve the diffusion of the precursors in the polymer matrix by removing diffusional barriers at the polymer surface. The HS, on the other hand, prolongs the exposure to the precursors, increasing the probability of forming germination sites, which will then grow to become islands.

A different pattern is observed for TS, and there is no direct correlation between the decreases in WCA and TS. PE48 shows a drastic decrease of TS only after 100 #cy both with and without HS. The difference from the bare membrane to the coated samples with or without HS is marginal for 25 and 50 #cy. A similar sudden suppression of TS is observed for PE83, this time appearing at 50 #cy. In this case, there is an improvement in TS when using 25 #cy and HS (down to 40%), but it is still incomplete. The number of required cycles to suppress TS is different for PE48 and PE83–100 and 50 #cy. Since the membranes are made of the same polymer, the difference must depend on the structural morphology.

Knowing from the WCA measurements that a total surface coverage is obtained after 50 #cy for PE48 but the suppression of shrinkage is reached only at 100 #cy, we hypothesize that a minimum threshold thickness of the AlO*_x_* ceramic shell around the polymer matrix must be reached to prevent shrinkage. From a mechanical perspective, the melting polymer exerts compressive stress on the shell. The coating can withstand this stress if it is below the fracture strength of the ceramic layer. Following the definition of stress as the force divided by the surface, we assume that increasing the thickness of the coating helps decrease the compressive test imposed on the shell’s cross section. The threshold thickness is reached once the shell is thick enough to resist the compressive stress of the PE membrane without collapsing. The latter could strongly depend on the morphology and on the mass of the polymer matrix. PE83 has a lower surface density compared to that of PE48; in fact, for an approximate PE density of 0.9 g/cm^3^,^33^ the surface densities of PE48 and PE83, based on their porosity and thickness, are 0.7 and 0.3 mg/cm^2^, respectively. The twofold difference seems to be correlated with the number of ALD cycles necessary to suppress the TS.

**Figure 4:**
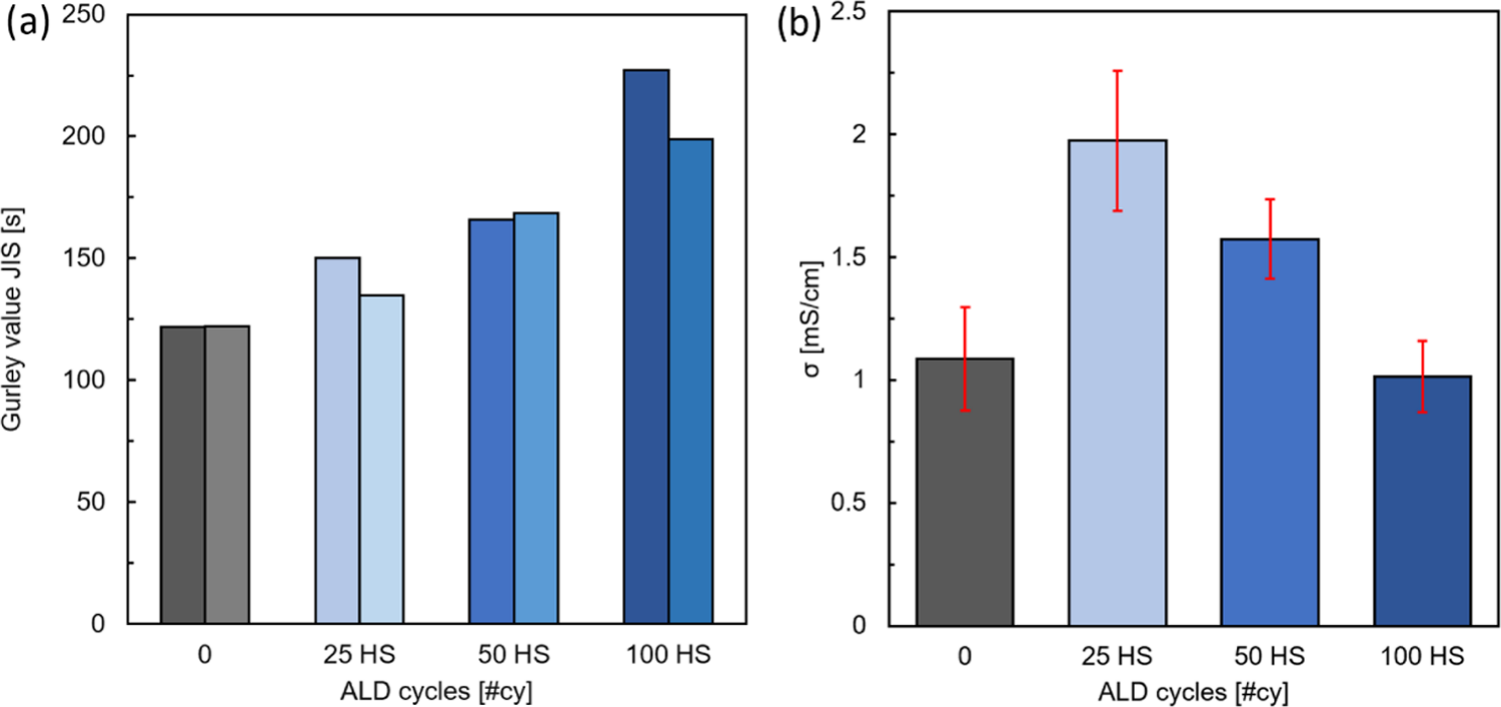
(a) Gurley value for the two samples of PE48 separator for an increasing number of ALD cycles and (b) ionic conductivity of the same samples in symmetrical stainless steel coin cells. EIS data is an average from two coin cells tested eight times each.

After effectively suppressing TS, the influence of the AlO*_x_* coating on the membrane’s permeability and ionic conductivity was studied to assess the electrochemical properties of the coated separators; for this purpose, the PE48 separator was selected instead of PE83, which is not robust enough for battery applications. Figure 4a shows the Gurley value of the PE48 membranes with an increasing number of ALD cycles using an HS. Figure 4b displays the ionic conductivity σ of the coated separators. The Gurley value of PE48 increases with the increasing number of ALD cycles. Given its inverse proportionality with the permeability parameter of the separator, it is concluded that the thicker the AlO*_x_* coating, the lower the separator’s permeability. It is hypothesized that the ceramic shell reduces the porosity, thus hindering the gas flow through the membrane. This observation could be a consequence of pore narrowing and, eventually, clogging of the lower fraction of the pore size distribution. A different trend is found when comparing the ionic conductivity of the separators: σ initially increases from 1 to 2 mS/cm after 25 #cy with HS and then decreases with the increasing number of cycles, returning to the initial value of 1 mS/cm after 100 #cy with HS. This trend could be explained by a combination of improved wettability and decreased permeability, both due to the increased thickness of the AlO*_x_* coating. The initial increase in ionic conductivity results from the improvement of the surface interaction with the liquid electrolytes: polyolefin materials have high contact angle with polar solvents, which slows down the separator impregnation and prevents the wetting of the smaller pores. We hypothesize that the ceramic shell reduces the liquid–solid contact angle and allows the electrolyte solution to fill a larger fraction of pores, thus improving the ionic conductivity. With the increasing ALD cycles, the smaller pores are clogged first, hence their contribution to the ion transport is lost and the conductivity decreases to the initial value.

**Figure 5:**
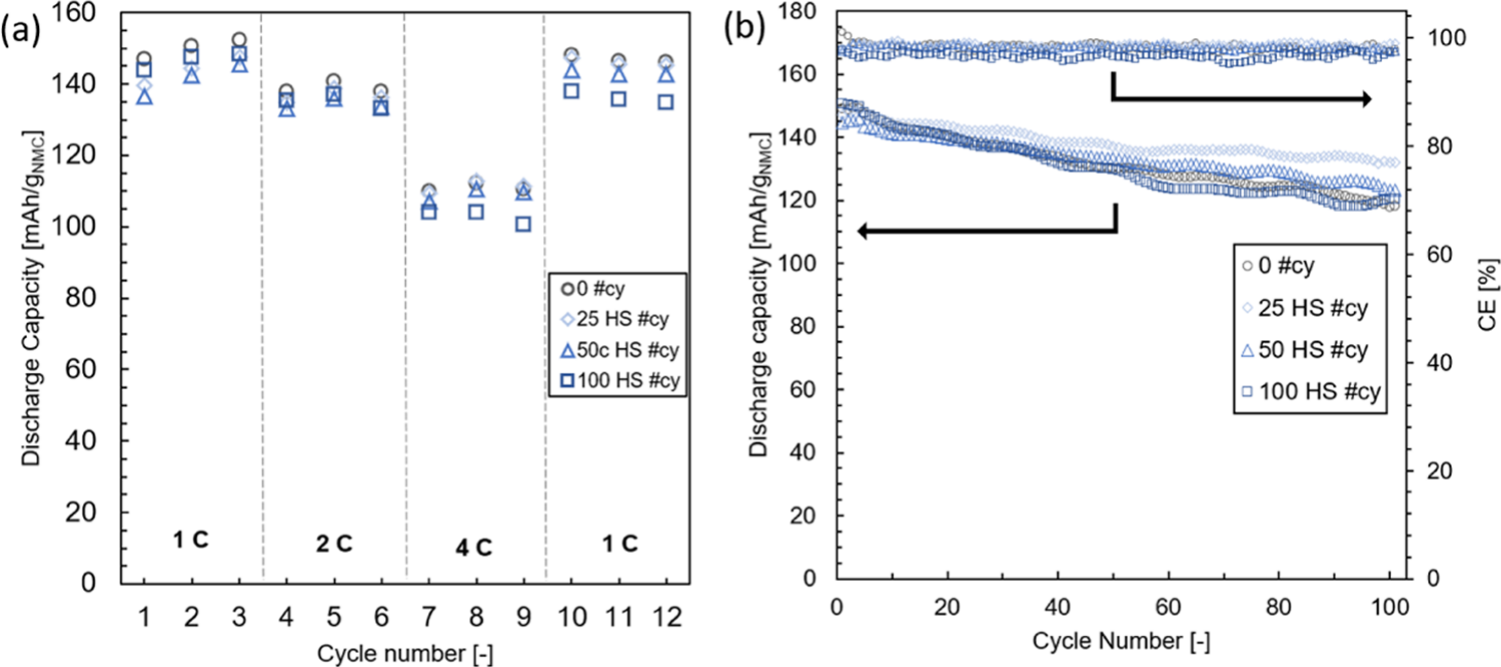
(a) Electrochemical performance of NMC532/graphite full cells in 1M LiPF6 in a mixture of EC/DMC/DEC 1:1:1 using PE48 bare and with 25, 50, and 100 #cy. Discharge capacity averaged from two batteries at different rates after two forming cycles at 0.2C and (b) subsequent capacity fading performance and Coulombic efficiency at 1C charge and 1C discharge.

The ionic conductivity trends presented in Figure 4b are reinforced by the cycling data of coin cells assembled in a full-cell configuration. Figure 5a displays the rate capability of graphite/NMC532 cells using PE48 separators and AlO*_x_* ALD coatings with HS and with an increasing number of cycles. Following two forming cycles at 0.2C rate (not shown), the first three cycles at 1C do not display a significant difference in discharge capacity between coated or uncoated separators. Increasing the discharge rate to 2C and subsequently 4C leads to a ∼5% drop in discharge capacity for the 100 #cy separator. The 25 and 50 #cy samples instead overlap with the bare PE48. Finally, the cells undergo three more cycles at 1C charge and discharge. A permanent capacity fading is observed for the 100 #cy samples, indicating that permanent damage was inflicted to the battery electrodes for the thicker AlO*_x_* coating. Since the electrodes are NMC532 and graphite, the operating C-rates are limited to 1C for charging and 4C for discharging. By testing the coin cell at these rates, the electrodes are pushed to their operational limit. For a high number of ALD cycles, hindering battery performance was previously reported.^17^ The same is observed for the 350 #cy Trilayer separator, displayed in Figure S4 in the Supporting Information. This was attributed to an increase in internal resistance due to pore clogging.

The ALD-coated separators are then compared based on discharge capacity fading of graphite/NMC532 cells cycled at 1C-rate both in charge and discharge (Figure 5b). The resulting curves are obtained by averaging two cells per condition. It is observed that cells assembled with the bare, 50, and 100 #cy separators have overlapping discharge capacities. The cells made with the 25 #cy samples instead have a smaller fading rate and therefore demonstrate a 10% higher discharge capacity relative to the bare separators curve after 100 testing cycles. This finding correlates with the higher ionic conductivity measured with the symmetrical cells (Figure 4b). The higher performance of the 25 #cy separator can be explained by the improved ion transport through the porous structure, which, thanks to the ceramic shell, is better wetted than the bare separator. The core–shell functionalization not only improves the thermomechanical integrity of the separator but also enhances the electrochemical performance. The impact on the membrane properties can be tuned depending on the ALD recipe, mainly with the number of deposition cycles. With 25 #cy, the structural failure of the melting polymer above 140 °C is reduced from ∼100 to 40% shrinkage, while the discharge capacity retention is 10% higher than its bare counterpart. With 100 #cy, TS is completely suppressed and no negative impacts on the battery performance are observed at 1C cycling; however, when discharging at high C-rates, permanent damage can be induced. This reveals a trade-off relationship between mechanical properties and electrochemical performance: composite separators can mitigate the risk of thermal runaway and improve cell performance, but increasing the coating thickness leads to permanent damage when discharging at high C-rates. It is hypothesized that further optimization of the ALD process could lead to both TS suppression and enhancement of capacity retention.

For polyolefin-based separators, the total suppression of TS is achieved only with a core–shell functionalization, as in literature, similar results are only achieved by a wet coating of expensive high-performance polymers such as polyimide or aramids. The better thermomechanical integrity of the ALD-coated PE separators (Figure 3) is a significant step toward thermal runaway mitigation, making the separator more resilient to both mechanical and thermal abuse. This can be achieved by selecting a separator that is well suited for ALD, mainly with higher porosity than the conventional Celgard separators. The advantage of composite designs with a core–shell functionalization, when compared to traditional wet coating techniques, is that the coating has a negligible contribution to the structural dimension of the separator (the thickness of the shell, supposedly <10 nm, is 3 orders of magnitude smaller than that of a ceramic particle coating on top of the separators). ALD coating thus allows thinner, safe separators, which, in turn, increase the battery’s energy density.

## Conclusions

In this work, it was found that AlO*_x_* ALD performed on polyolefin-based separators provides significant improvement in thermomechanical integrity and surface interaction with liquid electrolytes. Moreover, depending on the separator material (PP/PE or PE) and its porosity, a different number of cycles is needed to suppress thermal shrinkage. Optimization of the deposition process by pretreating the separators with a plasma step allows us to totally suppress thermal shrinkage at 100 deposition cycles for 15 μm PE separators of 48% porosity. The introduction of a hold step in the ALD cycle reduces the WCA of the coated separator by a factor of 2, reaching 38° after 25 deposition cycles. Symmetrical and full battery electrochemical characterizations show that the inorganic AlO*_x_* ALD coating doubles its ionic conductivity when wetted with conventional liquid electrolytes and extends the battery’s lifespan compared to an uncoated counterpart.

## Supporting Information Available

Extended material characterizations and cycling data (PDF)
