# Supplementary material for: Ultrathin ALD Aluminum Oxide Thin Films Suppress the Thermal Shrinkage of Battery Separator Membranes
Source: ACS Omega. 2022 Nov 30;7(49):45582–9. doi: 10.1021/acsomega.2c06318 (PMC9753167; doi:10.1021/acsomega.2c06318)
Supplement: Supplementary file 1 — ao2c06318_si_001.pdf [file ao2c06318_si_001.pdf]

Ultrathin ALD aluminum oxide thin-films suppress thermal shrinkage of battery separator membranes

Supporting information

Leonardo Pires da Veiga [a], leonardo.piresdaveiga@csem.ch
Colin Jeanguenat [a,1], colin.jeanguenat@csem.ch
Fabiana Lisco [b], fabiana.lisco@epfl.ch
Heng-Yu Li [a], hengyu.li@csem.ch
Sylvain Nicolay [a], sylvain.nicolay@csem.ch[2]
Christophe Ballif [b], christophe.ballif@epfl.ch
Andrea Ingenito [a], andrea.ingenito@csem.ch
Juan Jose Diaz Leon [a], juan.diaz@csem.ch

a Centre Suisse d'Electronique et de Microtechnique SA, Sustainable Energy Center, Neuchâtel, Switzerland

b Ecole Polytechnique Fédérale de Lausanne, PV-Lab, Neuchâtel, Switzerland

[1] Now at: The Laboratory for Molecular Engineering of Optoelectronic Nanomaterials (LIMNO), EPFL, Lausanne, Switzerland.
[2] Now at: Institut Interdisciplinaire d'Innovation Technologique (3IT), Sherbrooke University, Sherbrooke, Canada

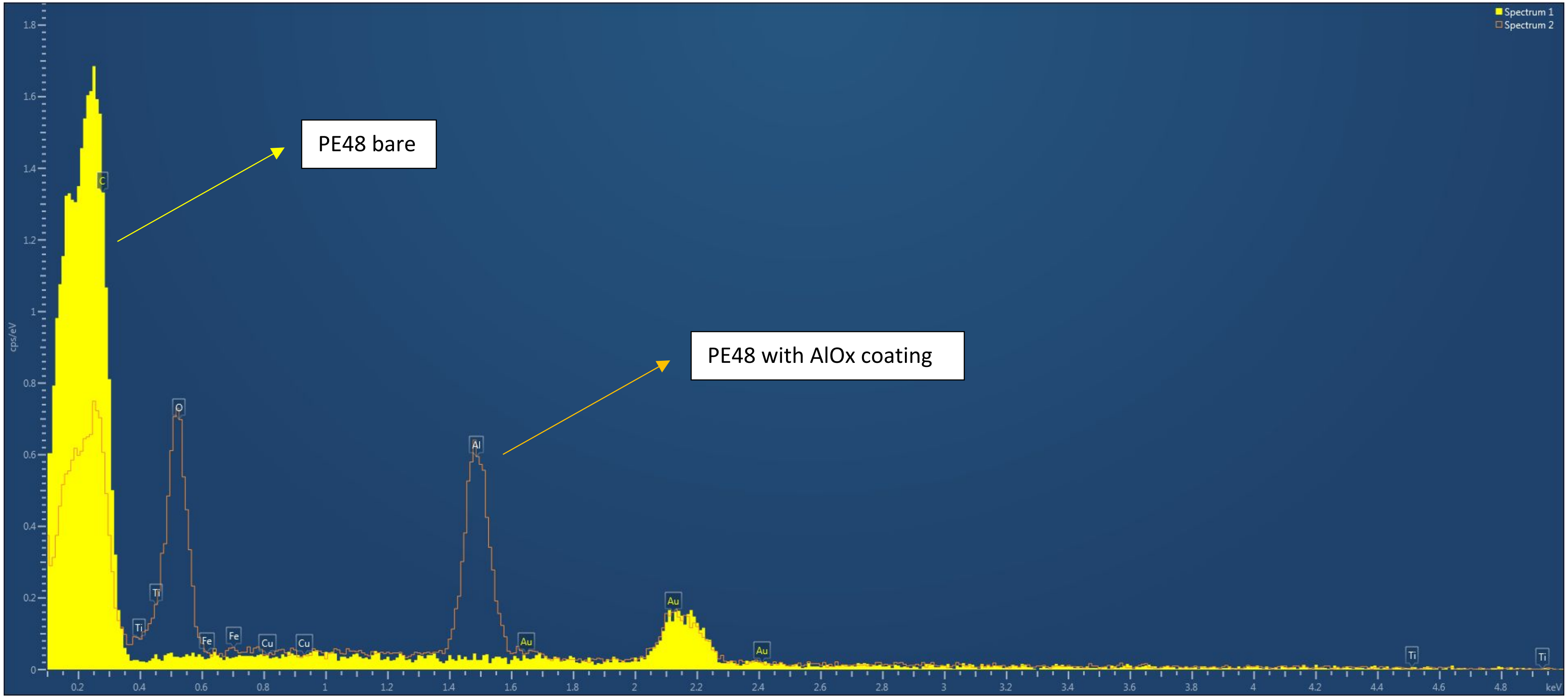

Figure S1 Energy dispersive X-ray spectroscopy of bare (yellow filling) and AlOx ALD coated (empty filling) polyethylene separator of 48% porosity. The coated sample shows the appearance of Oxygen and Aluminum characteristic peaks.

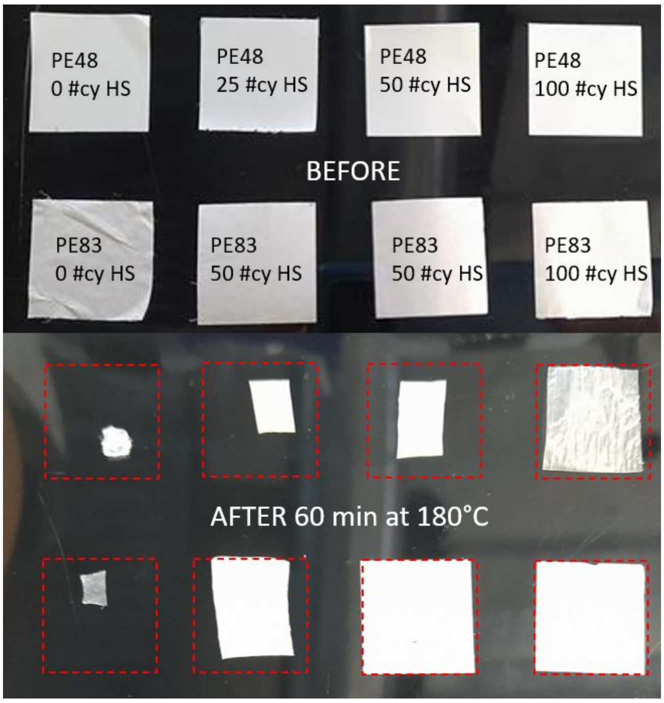

Figure S2 Thermal shrinkage of PE48 and PE83 membranes before and after 60 min at 180°C. The membranes are 2cmx2cm. The thermal shrinkage of PE48 100 #cy HS is measured after flattening (see wrinkles in picture).

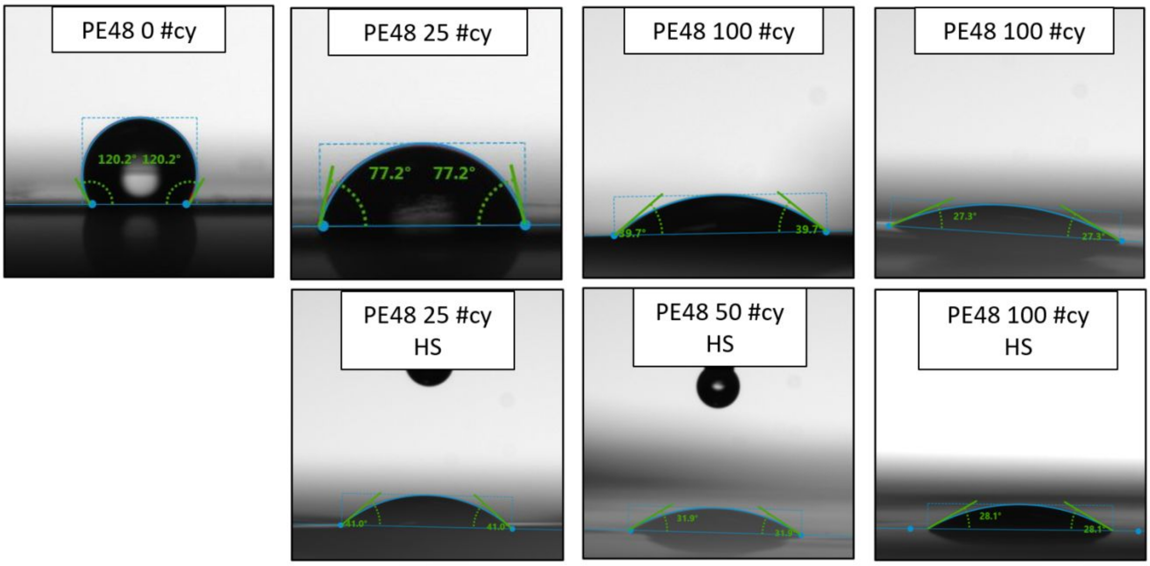

Figure S3 Water contact angle of PE48 membranes.

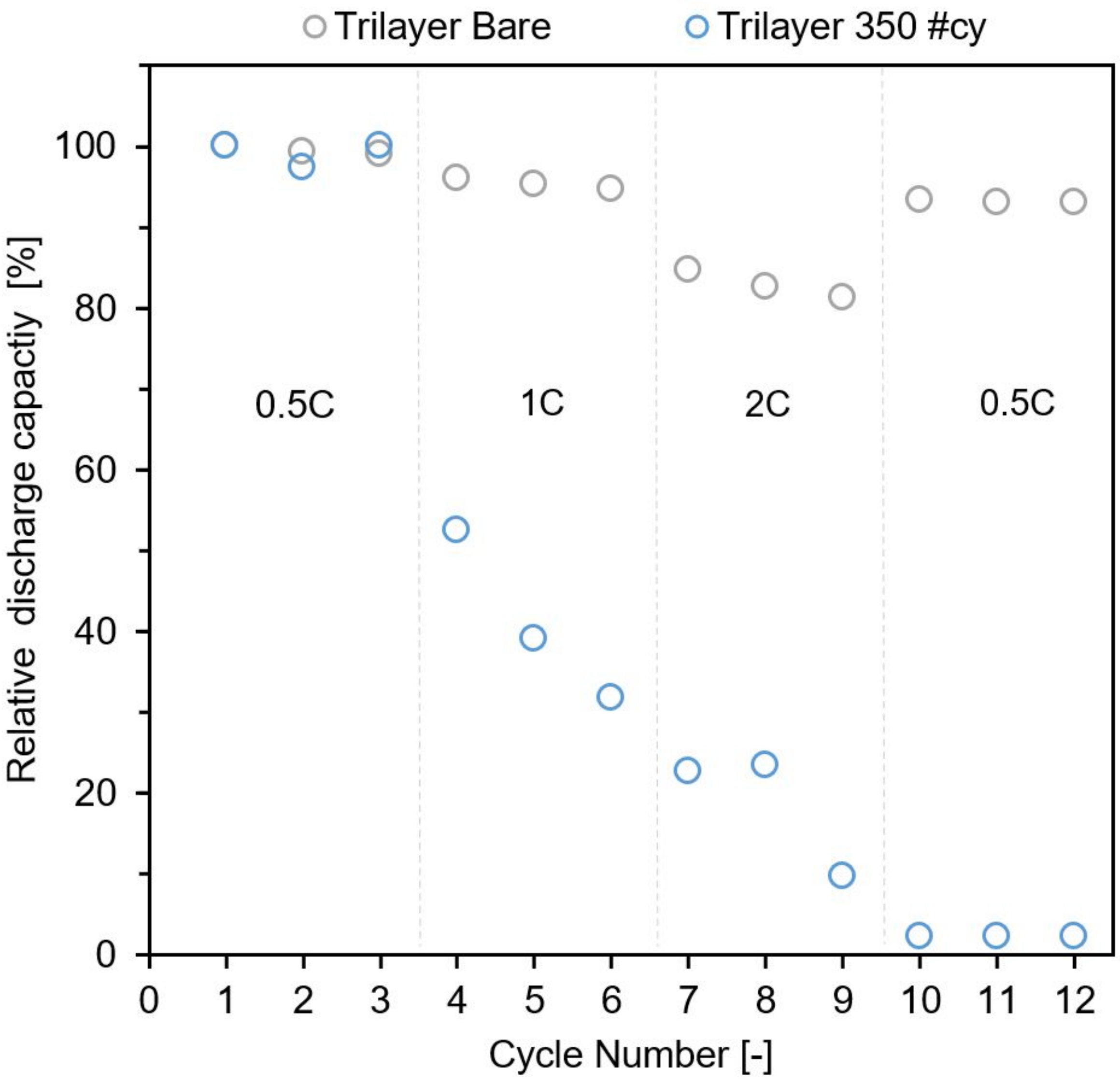

Figure S4 Electrochemical performance of NMC532/graphite full cells in 1M LiPF6 in mixture of EC/DMC/DEC 1:1:1 using Trilayer bare and with 350 ALD cycles. Discharge capacity averaged from two batteries at different rates after two forming cycles at 0.2C.
